# A novel risk classification system for 30-day mortality in children undergoing surgery

**DOI:** 10.1371/journal.pone.0191176

**Published:** 2018-01-19

**Authors:** Oguz Akbilgic, Max R. Langham, Arianne I. Walter, Tamekia L. Jones, Eunice Y. Huang, Robert L. Davis

**Affiliations:** 1 University of Tennessee Health Science Center-Oak Ridge National Laboratory Center for Biomedical Informatics, Memphis, Tennessee, United States of America; 2 Department of Preventive Medicine, University of Tennessee Health Science Center, Memphis, Tennessee, United States of America; 3 Department of Surgery, University of Tennessee Health Science Center, Memphis, Tennessee, United States of America; 4 Department of Pediatrics, University of Tennessee Health Science Center, Memphis, Tennessee, United States of America; 5 Children's Foundation Research Institute, Le Bonheur Children's Hospital, Memphis, Tennessee, United States of America; Boston Children's Hospital / Harvard Medical School, UNITED STATES

## Abstract

A simple, objective and accurate way of grouping children undergoing surgery into clinically relevant risk groups is needed. The purpose of this study, is to develop and validate a preoperative risk classification system for postsurgical 30-day mortality for children undergoing a wide variety of operations. The National Surgical Quality Improvement Project-Pediatric participant use file data for calendar years 2012–2014 was analyzed to determine preoperative variables most associated with death within 30 days of operation (D30). Risk groups were created using classification tree analysis based on these preoperative variables. The resulting risk groups were validated using 2015 data, and applied to neonates and higher risk CPT codes to determine validity in high-risk subpopulations. A five-level risk classification was found to be most accurate. The preoperative need for ventilation, oxygen support, inotropic support, sepsis, the need for emergent surgery and a do not resuscitate order defined non-overlapping groups with observed rates of D30 that vary from 0.075% (Very Low Risk) to 38.6% (Very High Risk). When CPT codes where death was never observed are eliminated or when the system is applied to neonates, the groupings remained predictive of death in an ordinal manner.

## Introduction

Over the past 30 years there has been a dramatic decline in surgical mortality for children and adults in the United States [1, 2]. The American College of Surgeons National Surgical Quality Improvement Program-Pediatric (NSQIP-Pediatric) dataset provides information on both inpatient and outpatient operations on children from birth to 18 years of age [3]. The 30-day mortality after operations in this dataset is approximately 0.3%, [4]. Since over 5 million infants and children undergo a surgical procedure in the United States annually [5], even a low mortality rate still places thousands of children at risk for death after surgery. The safety of surgery [6, 7,8] and anesthesia [9,10] in these children has been the topic of a number of recent studies, and is a quality measure that is increasingly important to payors, professional organizations, and patient advocacy groups.

Rare events, such as death after surgery in children, are difficult to accurately predict and no common method has been adopted. The American Society of Anesthesiologist’s Physical Status score (ASA-PS) is the most widely used risk stratification system for children undergoing surgery. Within the United States it is used to plan and adjust preoperative, intraoperative and postoperative care, and is important in adjusting healthcare billing [11]. Poor interrater reliability of ASA-PS has been reported in children [12]. Nonetheless, the ASA-PS score has consistently been an important part of mortality models published by NSQIP-Pediatric and is used in that organizations online risk calculator [13]. The logistic regression mortality models used for this calculator have large standard errors, and have not been able to separate contributing hospitals, or participating surgeons based on risk adjusted mortality. Other scoring systems such as the NARCO-SS (neurological, airway, respiratory, cardiovascular, other-surgical severity) have been shown to have better discrimination for composite adverse events but not for prediction of perioperative outcomes in individual patients [14].

To address the limitations of the existing risk stratification systems for children in surgery, a simple, objective, and accurate way of grouping children into clinically relevant risk groups is therefore needed. Such a system has the potential to improve individual patient care, clinical outcomes research, and clinical quality improvement projects. An ideal risk stratification would 1) be based on objective information that is easy to obtain, 2) be applicable across types of operations, and 3) work well for all age groups and medical conditions found in children.

The purpose of this study is to develop and validate a preoperative surgical mortality risk assessment of postsurgical 30-day mortality in children. The performance of this system was compared with a “best” logistic regression (LR) model and its accuracy tested in a subset of operations where a non-zero mortality rate was observed, and in neonates.

## Materials and methods

### Data set

The Pedi-PUF is a Health Insurance Portability and Accountability Act (HIPAA)-compliant data file containing cases submitted to NSQIP-Pediatric. The Pedi-PUF dataset contains patient-level, aggregate data and does not identify hospitals, healthcare providers, or patients. The intended purpose of the file is to provide researchers at participating sites with a data resource to investigate and advance quality of care [15]. For this study, we used Pedi-PUF data covering the years 2012–2015. The Pedi-PUF dataset contains over 300 perioperative variables that include operation type, defined by the primary CPT code assigned by the operating surgeon, demographic variables, data on the preoperative state of the child, including preexisting co-morbid conditions, medications, the results of laboratory tests, intraoperative data including length of the operation, blood loss and specific intraoperative and postoperative occurrences. Dillon et al have published details of data acquisition for the Pedi-PUF and NSQIP-Pediatrics [16]. The University of Tennessee Institutional Review Board considered this work exempt.

### Variables used

The outcome variable used in this study was death within 30 days following surgery (D30). Independent variables include gender, race, age, neonatal status, 48 preoperative variables, and the CPT code of the primary operation performed. Race was redefined as White, African-American or Other. This was done because of the small number of non-White, non-African American children in the PUF. All data were subjected to logic checks. Height, weight, and body mass index (BMI) had the highest rate of missing data or data outside of logic boundaries (i.e. age< 1 year with weight >25kg). These variables were excluded from analyses. The definition of neonate varied across the period of this study and for our analysis, neonatal status was classified using the algorithm in S1 Table. A total of 107 cases had the status of neonate listed as “no”, while congenital malformations was listed as “yes, neonate<1500 grams.” These cases were excluded due to this logical inconsistency (32 in 2012, 32 in 2013, and 43 in 2014, respectively). There were no deaths within 30 days following these cases. The categorical variable cardiac risk factor was converted into 4 dichotomous variables (none, minor, major, severe) and case type was converted to three dichotomous variables (elective, urgent, emergent). We elected to exclude the American Society for Anesthesia (ASA) score since this is itself a risk stratification score and has been shown to be collinear with many of the other preoperative variables in the data set.

### Tests on restricted data bases

CPT codes were ordered by frequency of observed D30. All CPT codes that had at least 1 death observed were arbitrarily assigned as high risk operations, while those having no observed mortality were considered low risk. Each case in the high-risk group was then assigned a risk strata and the observed mortality for the risk strata recalculated and plotted. All neonates were similarly assigned to a risk group as were patients who were not neonates. Again, observed mortality was calculated for each risk strata and plotted.

### Theory/calculation

#### Classification tree analysis

Classification and regression tree (CART) analysis [17] is a non-parametric statistical method that divides data into groups that have different values for one variable where this variable’s value results in the largest possible separation of the outcome variable (in this study, death within 30 days following surgery). The resulting two groups are then split in a similar fashion. This process is halted when preselected stopping criteria are reached [18]. Details of the method are easily obtained [19,20,21]. To determine the “best” classification tree we used all combinations of two of the three years of data (2012–2014) and tested the result using the 3^rd^ year of data. The sensitivity and specificity of each classification tree was calculated using the observed values of death within 30 days following surgery for each terminal node and summing the correct and incorrect classifications for all terminal nodes. The “best” candidate classification tree was defined as that tree with specificity >95% that had the highest sensitivity. The end product of this process was a set of stopping rules that maximized the classification accuracy of the model over each set of test data. A final classification tree was then constructed on all 2012–2014 data using these stopping rules.

#### Validation strategy

Both internal and external validation were performed in developing the risk classification. For internal validation, the PUF 2012–2014 data was split by year the operation was performed. A classification model was built on every possible combination of 2 years of data and tested on the remaining year. The goals of this step were to 1) arrive at stopping rules that did not over fit the data, and 2) to assess the generalized performance of the risk classification models. The consistency of the classification accuracy (in terms of specificity and sensitivity) from training to testing data were considered indicators of the generalizability of the classification models. Stopping rules that maximized the classification accuracy were then used to build a classification model using the entire PUF 2012–2014 dataset. The external validation step used this model to assign risk to every operation in the 2015 PUF. None of these operations had been used in any of the exploratory analysis. The observed mortality by assigned risk classification was measured for ordinal increase in the observed mortality and whether the observed mortality fit within the 95% confidence interval of the model built on 2012–2014 data.

#### Logistic regression

We compared the variables chosen for the best classification tree model and its classification accuracy to a stepwise logistic regression model derived from analysis of the same data. The same 55 preoperative variables used in the classification tree analysis were used in a stepwise logistic regression model on the Pedi-PUF 2012–2014 data with a cross validation strategy identical to that used for the classification tree model. Variables included in the model were checked to insure multi-collinearity between variables did not exist. Finally, we fit a logistic regression model limited to the variables that were identified as statistically significant in the classification tree model in order to assess its performance on validation data from 2015. We performed our analysis using Matlab version R2015a and SPSS 22 software. The University of Tennessee Institutional Review Board considered this work exempt.

## Results

The Pedi-PUF dataset includes 51,008 surgical operations performed in 2012 at 50 participating hospitals, 63,387 surgical operations performed in 2013 at 56 hospitals, 68,838 surgical operations performed in 2014 at 64 hospitals, and 84,506 surgical operations performed in 2015 at 80 hospitals. After data cleaning and logic checks, the 2012–2014 dataset used to build the model contained 183,123 operations on children less than 18 years of age. There were 621 children who died within 30 days following surgery: 178 out of 50,975 surgeries (0.35%) in 2012; 209 out of 63,354 surgeries (0.33%) from 2013, and 234 out of 68,794 surgeries (0.34%) from 2014. The overall observed operative mortality rate from 2012–2014 was 0.0034 (0.34%). The mortality for 2015 was 0.37%, slightly higher than any of the previous year’s.

### Univariate analysis

Risk factors for data from 2012–2014 were categorized in three groups: demographics, comorbidities, and preoperative treatments. Univariate statistics for each of these three groupings are presented in Tables 1, 2 and 3. African American children had 2.22 (95% confidence interval (CI): 1.83–2.70) times risk of death in the 30 days following surgery compared to white children (Table 1). Children born premature had approximately four-fold higher risk, while neonates undergoing surgery were at over twenty-fold higher risk to die within 30 days following surgery than non-neonates. The median age of children who died (35 days) was significantly less than the median age of those who survived (2344 days) (Mann Whitney U Test (p<0.01)).

**Table 1:** Patient characteristics and risk for death after surgery.

Risk Factors		Death within 30 days of operation		Odds Ratio (95% Confidence Interval)
		Yes	No	
Neonate	Yes	384	10,771	25.8 (21.95–30.41)
	No	237	171,731	
Premature birth	Yes	347	42,896	4.12 (3.52–4.83)
	No	274	139,606	
Race	Black	139	23,263	2.22 (1.83–2.70)
	White	349	130,018	
Sex	Male	332	104,674	0.85 (0.73–1.00)
	Female	289	77,827	

**Table 2:** Comorbidities and risk for death after surgery.

Risk Factors		Death within 30 days of operation		Odds Ratio (95% Confidence Interval)
		Yes	No	
Acute Renal failure	Yes	28	319	26.97 (18.17–40.01)
	No	593	182,183	
Bleeding disorders	Yes	78	1,122	23.22 (18.19–29.65)
	No	543	181,380	
Hematologic Disorder	Yes	202	5,543	15.39 (12.99–18.24)
	No	419	176,959	
Pneumonia	Yes	31	652	14.66 (10.13–21.20)
	No	590	181,850	
Cardiac Risk Factor*	No	309	166,061	0.10 (0.08–0.12)
	Minor	99	9,416	3.49 (2.81–4.33)
	Major	157	5,756	10.39 (8.65–12.48)
	Severe	56	1,269	14.15 (10.70–18.73)
Intraventricular Hemorrhage (IVH) Grade	Yes	93	3,775	8.34 (6.67–10.42)
	No	528	178,727	
Severe COPD	Yes	136	6,249	7.91 (6.53–9.58)
	No	485	176,253	
Esophageal varices	Yes	365	32,738	6.52 (5.56–7.66)
	No	256	149,764	
Biliary/Liver/Pancreatic Disease	Yes	74	3,935	6.14 (4.81–8.84)
	No	547	178,567	
Systemic Sepsis	Yes	177	11,948	5.69 (4.78–6.78)
	No	444	170,544	
Structural Pulmonary/Airway Abnormalities	Yes	133	10,165	5.12 (4.24–6.17)
	No	477	172,337	
CVA/Stroke or traumatic/ acquired brain injury with resulting neurological deficit	Yes	59	4,131	4.53 (3.46–5.94)
	No	562	178,371	
Previous cardiac surgery	Yes	81	5,864	4.52 (3.57–5.72)
	No	540	176,638	
Open wound/wound infection	Yes	42	2,970	4.39 (3.20–6.01)
	No	579	179,531	
Immune Disease / Immunosuppressant Use	Yes	26	1,884	4.19 (2.82–6.22)
	No	595	180,618	
Childhood Malignancy	Yes	58	5,204	3.51 (2.68–4.61)
	No	563	177,298	
Tracheostomy	Yes	21	1,962	3.22 (2.08–4.99)
	No	600	180,540	
Seizure	Yes	81	8,868	2.94 (2.32–3.71)
	No	540	173,634	
>10% loss body weight in last 6 months	Yes	53	5,801	2.84 (2.14–3.77)
	No	568	176,701	
Structural CNS Abnormality	Yes	150	21,019	2.45 (2.04–2.95
	No	471	161,483	
Diabetes mellitus	Yes	5	718	2.06 (0.85–4.97)
	No	616	181,784	
Neuromuscular Disorder	Yes	54	8,840	1.87 (1.41–2.48)
	No	567	173,662	
Congenital Malformation	Yes	241	52,941	1.55 (1.32–1.83)
	No	380	129,561	
Developmental delay	Yes	104	23,855	1.34 (1.08–1.65)
	No	517	158,647	
Cerebral Palsy	Yes	25	6,657	1.11 (0.74–1.65)
	No	596	175,845	
Cystic Fibrosis	Yes	2	549	1.07 (0.27–4.30)
	No	619	181,953	
History of Asthma	Yes	18	11,047	0.46 (0.29–0.74)
	No	603	171,455	

* For Cardiac Risk Factor variable, the odds ratios are calculated for each category compared to all of the other categories combined.

**Table 3:** Preoperative therapy and risk for death after surgery.

Risk Factors		Death within 30 days of operation		Odds Ratio (95% Confidence Interval)
		Yes	No	
Previous CPR within 7 days prior to surgery	Yes	71	194	121.31 (91.28–161.23)
	No	550	182,308	
Unconscious, or postures to painful stimuli, or is unresponsive to all stimuli entering surgery (COMA)	Yes	8	22	108.25 (48.01–244.01)
	No	613	182,480	
Do-not-resuscitate order	Yes	24	84	85.27 (53.86–135.02)
	No	597	182,416	
Inotropic Support	Yes	219	1,167	84.65 (71.10–100.78)
	No	402	181,335	
Ventilator assisted respiration at any time during the 48 hours preceding surgery.	Yes	429	4,749	83.63 (70.37–99.39)
	No	192	177,753	
Oxygen support at the time of surgery	Yes	381	5,882	48.18 (40.91–56.74)
	No	240	176,680	
Transfusion of > = 1 unit of whole / packed RBCs in 72 hours prior to surgery	Yes	186	1,769	43.69 (46.56–52.20)
	No	435	180,733	
Nutrition Support	Yes	401	14,001	21.12 (17.90–24.91)
	No	220	168,001	
Radiotherapy for malignancy in last 90 days	Yes	7	148	14.05 (6.56–30.10)
	No	641	182,354	
Steroid use for chronic condition	Yes	152	4,518	12.77 (10.61–15.37)
	No	469	177,984	
Dialysis (pre-op)	Yes	25	557	13.70 (9.11–20.62)
	No	596	181,945	
Bone Marrow Transplant	Yes	14	334	12.58 (7.33–21.60)
	No	667	182,168	
Chemotherapy for malignancy in < = 30 days pre-op	Yes	25	994	7.66 (5.11–11.48)
	No	596	181,508	
Case Status*	Elective	213	133,781	0.19 (0.16–0.23)
	Emergent	321	30,444	5.34 (4.56–6.26)
	Urgent	87	18,277	1.46; 1.17–1.84)
Organ Transplant	Yes	4	444	2.66 (0.99–7.14)
	No	617	182,058	
Outpatient	Yes	15	79,271	0.03 (0.02–0.05)
	No	606	103,231	

* For Cardiac Risk Factor variable, the odds ratios are calculated for each category compared to all of the other categories combined.

The absence of cardiac risk factors and the presence of asthma were associated with a decreased risk of death in the 30 days following surgery (Table 2). The presence of diabetes mellitus, cerebral palsy or cystic fibrosis had no impact on risk of death after surgery. The remaining risk factors all were associated with a statistically significant increase in death after surgery. Some conditions created a dramatic increase in risk, including a 25-fold increased risk for children with acute renal failure or with bleeding disorders, and a 15-fold increase for children with hematologic disorders, severe cardiac risk factors or pneumonia.

Children requiring a variety of medical treatments or interventions prior to surgery were at significantly higher risk of death (Table 3). As might be expected, the risk was especially elevated for children with do-not-resuscitate status, who required assisted ventilation at any time during the 48 hours preceding surgery, who were unconscious, or who had required cardiopulmonary resuscitation (CPR) recently prior to surgery.

There were differences between the 2012–2014 data and the 2015 data. Mortality in 2015 was higher (0.37% vs 0.34%, p<0.01). The 2015 data contained a higher proportion of operations on African American patients (White: African American is 5.8 for 2012–2014 and 5.3 for 2015). None of these differences was considered clinically significant.

### Classification tree analysis

#### Stopping criteria and cross-validation

Using the approach outlined in the methods section, the highest generalizability and classification performance was obtained when the minimum number of cases in groups to be further split was 80, and the minimum number of cases in final groups was 40. The optimal tree was 3 branches deep. Classification trees were developed using these stopping rules on all combinations of two years of data and tested on the third year of data (S1 File). Performances of these classification tree models were consistent across the combinations of years used as training and test data suggesting that over-fitting was minimal.

#### Building a clinically relevant, validated risk stratification for death within 30 days following surgery

An optimal tree was created using all data from 2012–2014 (S1 Fig). Five risk groups were created by grouping terminal nodes with similar observed mortality rates. The final risk stratification is presented in Table 4 with risk estimates and confidence intervals based on data from 2012–2014, and observed mortality in the 2015 PUF file. This stratification creates discrete subgroups of children whose risk of death within 30 days following surgery varies from <1% to 38.6%, in 5 discrete risk levels. The Very Low risk group contains the vast majority (95.5%) of children who had none of the identified risk factors. Combining all PUF files there were 223 (24% of total) deaths within 30 days of operation after 254,165 operations (0.09%) in the Very Low risk group. This represents a four-fold lower risk than the average risk seen in the overall study population. The highest risk group (Very High Risk), contains children who were on ventilation, receiving inotropic support and whose surgery was classified as an Emergent Case. This group of 537 cases had a 440-fold higher risk than the baseline population (38.60%, 95% CI: 33.74%-43.46%) and included 22% of all deaths (207 out of 931) in the study population. The final model had a specificity of 95.7%, and a sensitivity = 78.9% with c-statistic = 0.89, and used 6 variables: preoperative need for ventilator, oxygen support, or inotropic support, preoperative presence of sepsis, emergent case status = ‘yes’, and do not resuscitate = ‘yes’.

**Table 4:** Surgical mortality risk groups for children.

Risk Group	Criteria	Risk %, 95%CI for 2012–2014 Data	Observed mortality rate (%) for 2015 data
**Very Low**	**Ventilator = ‘N’ AND Oxygen Support = ‘N’ AND DRN = ‘N’**	**0.075 [0.062–0.088]**	**0.116**
**Moderately Low**	**Ventilator = ‘N’ AND Oxygen Support = ‘Y’ AND Sepsis = ‘N’**	**1.347 [0.922–1.772]**	**1.324**
**Moderately High**	**Ventilator = ‘N’ AND Oxygen Support = ‘Y’ AND Sepsis = ‘Y’**	**3.299 [2.721–3.877]**	**2.914**
	**Ventilator = ‘Y’ AND Inotropic Support = ‘N’ AND Emergent Case = ‘Y’**		
**High**	**Ventilator = ‘Y’ AND Oxygen support = ‘N’ AND DNR = ‘Y’**	**12.552 [10.846–14.257]**	**10.644**
	**Ventilator = ‘Y’ AND Inotropic Support = ‘N’ and Emergent Case = ‘Y’**		
	**Ventilator = ‘Y’ AND Inotropic Support = ‘Y’ AND Emergent Case = ‘N’**		
**Very High**	**Ventilator = ‘Y’ AND Inotropic Support = ‘Y’ AND Emergent Case = ‘Y’**	**38.601 [33.744–43.458]**	**38.411**

**Fig 1:**
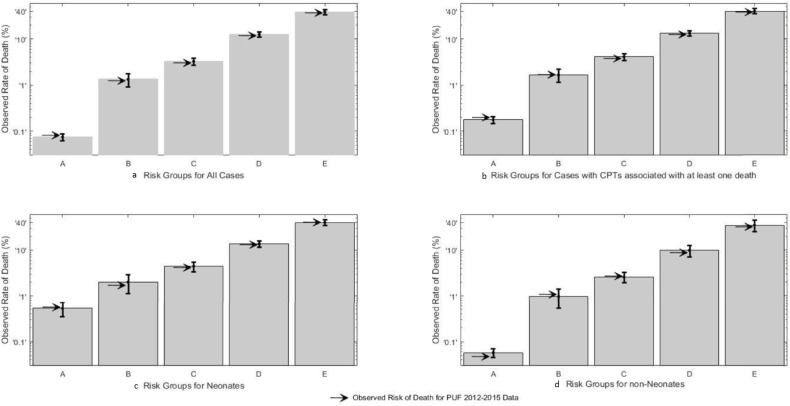
Observed mortality by risk group for sub groups. The observed mortality is plotted logarithmically by group. A = Very Low, B = Moderately Low, C = Moderately High, D = High, E = Very High. Each bar represents the estimate of the risk and confidence interval. The arrow is the observed rate for all patients from 2012–2015.

Each case in the 2015 Pedi-PUF had a risk group assigned. The 2015 observed mortality rate for three of the five risk groups fell within the 95% confidence intervals for those groups, as expected (Fig 1). The observed mortality for Very Low Risk group was 0.116%, above the confidence interval of the model [0.062%-0.088%], but well below the lower CI of the Moderately Low risk group [0.992%]. The observed mortality for the High Risk Group was slightly below the 95% CI but within the 99% CI [10.311–14.793] for the model.

### Logistic regression analysis

We next carried out logistic regression analysis on the same dataset to predict risk of death in the 30-days following surgery. The final model contained 31 variables (S2 Table). To compare the predictive accuracy of this model with the one created by classification tree analysis, we selected a cutoff value to classify patients according to their risk of death with specificity equal to that achieved by the classification tree model. With the specificity of the LR model set to 95.7%, (equivalent to that with classification tree), the sensitivity of the LR model was 82.3%, compared with sensitivity of 78.9% found by classification tree (an improvement of 3.4%). Since classification tree and stepwise LR resulted in two significantly different models in terms of model complexity, we implemented a second round of LR using nine variables that were selected in any stage of the classification tree building process. These variables were “Do-not-resuscitate order”, “Ventilator”, “Sepsis”, “Oxygen Support”, Inotropic Support”, “Emergent Case Type”, “Malignancy”, “Transfusion”, and “Age”. Table 5 shows that all the considered variables were significant in the last LR model and were more parsimonious in comparison to the LR model that was obtained through the stepwise method. The LR model using these nine variables showed a 78.4% sensitivity and a 95.7% specificity with c-statistic = 0.94, whereas the stepwise LR model with 31 variables showed 82.3% sensitivity, a 95.7% specificity, and had a c-statistic = 0.97.

**Table 5:** Parsimonious LR model built on variables selected by classification tree analysis.

	OR with 95% confidence interval	p-value
DNR	30.65 (16.32–57.58)	<0.01
Oxygen Support	2.620 (2.03–3.38)	<0.01
Sepsis	1.704 (1.35–2.16)	<0.01
Inotropic Support	4.440 (3.53–5.60)	<0.01
Transfusion	2.091 (1.65–2.65)	<0.01
Malignancy	5.731 (4.15–7.91)	<0.01
Emergent Case	2.330 (1.92–2.83)	<0.01
Age	1.000 (1.00–1.00)	<0.01
Ventilation	11.990 (9.08–15.84)	<0.01

### Risk stratification applied to clinical subgroups

The combined PUF data contained 267,289 operations and 647 primary CPT codes. D30 occurred at least once in 144 CPT codes that were termed “high risk”. D30 was never observed in 503 CPT codes designated “low risk.” Operative complexity was not associated with “high” risk operations. For instance, there were 22 deaths after 1,578 gastrostomy tube insertions (open or laparoscopic), but none after 4942 laparoscopic cholecystectomies. The observed mortality was plotted on a logarithmic scale by patient risk groups for all cases (Fig 1A), for 119,282 high-risk operations (Fig 1B), for 14,757 operations performed in neonates (Fig 1C), and for 252,532 operations performed in non-neonates (Fig 1D). In the overall analysis, and for each subset analysis the risk grouping resulted in ordinal increase in observed mortality from Very Low Risk to Very High Risk, without overlap of confidence intervals. In neonates, the difference between the observed mortality from Very Low to Very High was 100-fold; in non-neonates, the difference was nearly 1000-fold.

There were 148,007 “low risk” operations in the combined data set. In this group, operations coded with 33 CPT codes were done over 1000 times without mortality. Of the 78,160 operations in this subgroup there were only 605 or 0.8% that were not in the Very Low Risk Group. Interestingly 221 of these operations were a single CPT (22849; reinsertion of spinal fixation device). Most of these were on patients in Moderately Low or Moderately High risk groups. There were only 2 Very High risk operations in these 148,007 operations. Both were for incidental appendectomy (CPT code 44950).

## Discussion

We propose a novel five level risk classification model that predicts death within 30 days of surgery in children based on 6 variables. Very Low Risk children have a 0.075% risk of death within 30 days following surgery, while those in the High and Very High risk groups have a 12.5% and 38.6% risk of dying within 30 days after surgery, respectively. Children who prior to surgery had no need for ventilator or oxygen support, and who were not classified as ‘Do Not Resuscitate’, had less than a 1 in 1000 risk for dying during the 30 post-operative days despite undergoing a broad array of operations by a variety of specialties. The remaining children had more than a 10-fold higher risk, and some had more than a 400-fold increase in risk over baseline. This risk grouping is simple, and the data needed for assignment are unambiguous and easily measured. There was no overlap in the risk groups. This risk classification is fundamentally different than the results of a logistic regression, which provides a continuous numeric risk estimate between 0 (no chance of death) and 1 (certain death).

The results of the classification system and its implications are generally consistent with clinical experience. Other authors have published a higher probability of death after surgery in children requiring ventilator support and/or oxygen therapy [22, 23], who are hemodynamically unstable [24, 25], undergoing emergent operations, who are septic [26], have cancer [27], or who have a current do not resuscitate order [28]. There were several findings we find surprising or not in accord with the literature. The absence of “neonatal” status in the models, and presence of age in only one of the cross-validation classification trees differs from others who have noted neonates to have a much higher mortality than older children [29, 30, 31, 32]. The NSQIP-Pediatric data used in this analysis clearly shows a dramatic increase in mortality in neonates. Our work suggests this is most easily observable in neonates with no identified risk factors where the mortality for neonates remained less than 1% but was higher than non-neonates. Thus, in the United States, for NSQIP eligible patients, neonatal risk is primarily due to ventilator dependence, oxygen support, inotropic support, sepsis and emergent case status, and not specifically to age or maturity. The confirmation that the classification works when the data is restricted to neonates, and when neonates are excluded strengthens the concept that variables defining the risk grouping are more important than age, and can themselves account for much of the observed increase in D30 in neonates. In the absence of these risk factors neonates are at low risk of dying in NSQIP-Pediatric participating hospitals. Conversely in the presence of these risk factors, older children are at increased risk of death, at rates comparable to neonates with the same risk factors.

This dataset includes information on operations coded using 647 CPT codes. The classification system worked for 144 CPT codes associated with at least 1 death. The vast majority of children undergoing 503 CPT operations not associated with death were in Very Low Risk children. Other operations of similar complexity but performed more frequently in higher risk groups had at least 1 death observed. This finding has profound implications. NSQIP-Peds, like its adult counterpart is designed around the operation as defined by the CPT code. If adverse events other than D30 are more tightly associated with patient factors than with the operation, modifications of the NSQIP-Pediatric methodology may better inform future research on surgical quality and outcomes.

The classification tree analysis also suggests that relationships between risk factors are important. For instance, Group A includes children with appendicitis who have sepsis and are undergoing an emergent operation but who are actually at low risk of death within 30 days of the operation. For patients in other nodes the presence of sepsis or need for emergent surgery may be very important in their risk of dying after surgery.

Our paper has a number of strengths. First, the NSQIP-Pediatric data set is among the largest and most complete clinical data available on large numbers of children undergoing operations in the United States. Rigorous logic checking of the data was used to exclude variables of questionable value or whose data were suspect. Death within 30 days of surgery is unambiguous, easily measured and clinically important. By carefully excluding intraoperative and postoperative variables, only information available to surgeons and anesthesiologists before deciding to proceed with operations were used to develop these risk models. Definitions in the NSQIP-Pediatric data set are well defined, and changes in definitions published. Thus, we were able to construct a logically consistent neonatal variable over the range of dates included in the study. We also carefully excluded variables with high collinearity. For instance, many risk stratification methods, including reports published by NSQIP-Pediatric include the ASA classification as a variable [4, 33, 34]. ASA classification has been shown repeatedly to be highly associated with risk of death and other adverse events in children and in adults, but is dependent on the subjective opinion of an expert anesthesiologist [35, 36, 37]. We excluded it from our data set since our goal was to develop a risk stratification based on directly observable clinical variables.

Logistic regression (LR) is the most commonly used method to calculate risk in medicine. LR provides a continuous estimate of mortality between limits of [0, 1]. We sought instead to develop groupings of mortality risk. Cooper at al. [38] compared LR with Support Vector Machines, Classification Trees, and Random Forest for prediction of pediatric surgical morbidity using the Pedi-PUF 2012 dataset; classification tree methods performed as accurately as LR. Similarly, Demir [39] found that LR, classification tree analyses, Generalized Additive Models, and Multivariate Adaptive Regression Splines were equally accurate in predicting patients at risk of readmission. Neither used the CART models to propose specific risk groups. This study confirms that for death after surgery in children a CART model is simple and potentially easily applicable to the clinical realm. The finding that variables included in our final classification tree model are included in both a previous LR model generated on our hospital’s data [22] and the recently published NSQIP risk calculator [4] each using slightly different methods lead us to speculate that these variables represent a clinically relevant, robust core of information necessary for estimating death within 30 days following surgery among children undergoing these procedures.

Our study does have some weaknesses. These findings may not be generalizable to children undergoing operations with codes not captured, or under-sampled in the NSQIP-Pediatric data set. These include cardiac operations, organ transplants, and most operations for traumatic injuries. The analysis is also based on data that represents a nonrandom sample of children undergoing operation. The finding that African American children have a higher risk for D30 than white children deserves further study. A high rate of missing data for race in the data set makes it difficult to confidently draw conclusions from this observation, or to assess children not classified as African American or white. The difference in proportion of the two races in the 2015 PUF is one of potentially many explanations for the higher observed mortality in the 2015 PUF. The addition of more hospitals, that may have different mortality profiles, is another. Since the Pedi-PUF is de-identified and does not allow analysis of clustering within centers it is impossible to control for this possibility. The PUF also assumes independence between operations that may be performed on the same child. The lack of patient level identification makes it impossible to control for this factor.

We did not include surgical specialty as a variable in these studies. Thus, the risk stratification developed is applicable to all surgeons and operations sampled by NSQIP-Pediatric. Better risk estimations may or may not be possible by creating specialty or disease specific models but these studies will be hampered by much smaller sample sizes. Future research should focus on evaluating interactions between risk groups and CPT codes, specialties, and centers. To address these questions, datasets even larger than those studied here will be needed [40].

## Conclusions

A classification system based on preoperative status that separates children by their risk of dying in the 30 days after surgery is proposed. Using six variables, it is possible to accurately classify children into risk categories that differ more than 100-fold. Although we implemented both internal and external validation during model development, future work is needed to determine the clinical utility of this model as a clinical decision support system. In addition, the generalizability of risk models developed on NSQIP-Pediatric data needs to be tested on children undergoing procedures not captured in that database.

## Supporting information

S1 Table Neonate determination rule if neonate variable missing. (DOCX)

S2 Table Stepwise logistic regression results using 31 variables. (DOCX)

S1 File Figure A: Classification tree trained on 2013 and 2014 data and tested on 2012 data.
Figure B: Classification tree trained on 2012 and 2014 data and tested on 2013 data.
Figure C: Classification tree trained on 2012 and 2013 data and tested on 2014 data.
(DOCX)

S1 Fig Final classification tree built using the rule developed on training CTree’s and using all data from 2012–2014. (DOCX)
