# Supplementary material for: A novel risk classification system for 30-day mortality in children undergoing surgery
Source: PLoS One. 2018 Jan 19;13(1):e0191176. doi: 10.1371/journal.pone.0191176 (PMC5774754; doi:10.1371/journal.pone.0191176)
Supplement: S1 Table — (DOCX) [file pone.0191176.s001.docx]

**Supporting Information File 1**

**S1 Table**: **Neonate Determination Rule if Neonate Variable Missing**

|  | | | Neonate Decision |
| --- | --- | --- | --- |
| Age (days) | Premature Birth | Neonate |  |
| <=28 | No | Null | Yes |
| >28 | No | Null | No |
| <=140 | 24 completed weeks gestation | Null | Yes |
| >140 | 24 completed weeks gestation | Null | No |
| <=150 | Less than 24 completed weeks gestation | Null | Yes |
| >150 | Less than 24 completed weeks gestation | Null | No |
| <=119 | 27-28 completed weeks gestation | Null | Yes |
| >119 | 27-28 completed weeks gestation | Null | No |
| <=133 | 25-26 completed weeks gestation | Null | Yes |
| >133 | 25-26 completed weeks gestation | Null | No |
| <=63 | 35-36 completed weeks gestation | Null | Yes |
| >63 | 35-36 completed weeks gestation | Null | No |
| <=77 | 33-34 completed weeks gestation | Null | Yes |
| >77 | 33-34 completed weeks gestation | Null | No |
| <=91 | 31-32 completed weeks gestation | Null | Yes |
| >91 | 31-32 completed weeks gestation | Null | No |
| <=105 | 29-30 completed weeks gestation | Null | Yes |
| >105 | 29-30 completed weeks gestation | Null | No |
