# Supplementary material for: A novel risk classification system for 30-day mortality in children undergoing surgery
Source: PLoS One. 2018 Jan 19;13(1):e0191176. doi: 10.1371/journal.pone.0191176 (PMC5774754; doi:10.1371/journal.pone.0191176)
Supplement: S2 Table — (DOCX) [file pone.0191176.s002.docx]

**Supporting Information File 4**

S2 Table: Stepwise Logistic Regression results for LR performed with all preoperative variables

| **Variables in the Equation** | | B | Sig. | OR Exp(B) | 95% CI for OR | |
| --- | --- | --- | --- | --- | --- | --- |
|  |  |  |  |  | Lower | Upper |
|  | Outpatient | -1.510 | .000 | .221 | .129 | .379 |
|  | DNR | 2.595 | .000 | 13.393 | 7.203 | 24.899 |
|  | Premature | .299 | .005 | 1.348 | 1.096 | 1.658 |
|  | Ventilation | 1.607 | .000 | 4.988 | 3.790 | 6.564 |
|  | Bronchopulmonary dysplasia/chronic lung disease | .684 | .004 | 1.983 | 1.239 | 3.174 |
|  | History of cystic fibrosis | -.271 | .029 | .762 | .598 | .972 |
|  | Oxygen Support | .600 | .000 | 1.822 | 1.435 | 2.314 |
|  | Liver pancreatic, biliary disease | .444 | .006 | 1.558 | 1.138 | 2.134 |
|  | Major Cardiac Risk Factor | .318 | .008 | 1.374 | 1.086 | 1.739 |
|  | Severe Cardiac Risk Factor | 1.433 | .000 | 4.191 | 2.921 | 6.012 |
|  | Renal Failure | .580 | .027 | 1.786 | 1.069 | 2.982 |
|  | COMA | 1.633 | .009 | 5.119 | 1.498 | 17.496 |
|  | Seizure | .476 | .003 | 1.609 | 1.176 | 2.204 |
|  | Cerebral Palsy | .585 | .020 | 1.796 | 1.097 | 2.939 |
|  | Structural Pulmonary/Airway (SPA) Abnormalities | .441 | .000 | 1.554 | 1.239 | 1.950 |
|  | Neuromuscular Disease | .379 | .029 | 1.460 | 1.040 | 2.050 |
|  | Bone Marrow Transplant | 1.831 | .000 | 6.242 | 3.003 | 12.976 |
|  | Current wound infection | -.450 | .017 | .638 | .441 | .923 |
|  | Nutrition Support | .471 | .000 | 1.602 | 1.276 | 2.010 |
|  | Hemodisorder | .502 | .000 | 1.652 | 1.323 | 2.064 |
|  | Radiotherapy | 1.341 | .009 | 3.821 | 1.390 | 10.503 |
|  | Sepsis | .483 | .000 | 1.621 | 1.271 | 2.066 |
|  | Inotropic Support | 1.257 | .000 | 3.514 | 2.766 | 4.464 |
|  | CPR | 1.068 | .000 | 2.909 | 2.021 | 4.187 |
|  | Transfusion | .389 | .002 | 1.476 | 1.157 | 1.884 |
|  | Malignancy | 1.557 | .000 | 4.744 | 3.278 | 6.866 |
|  | Emergent Case | .993 | .000 | 2.698 | 2.158 | 3.374 |
|  | Urgent Case | .285 | .044 | 1.330 | 1.008 | 1.756 |
|  | Neonate | .826 | .000 | 2.284 | 1.735 | 3.006 |
|  | Age | .000 | .000 | 1.000 | 1.000 | 1.000 |
|  | Constant | -7.120 | .000 | .001 |  |  |
