# Supplementary material for: A novel risk classification system for 30-day mortality in children undergoing surgery
Source: PLoS One. 2018 Jan 19;13(1):e0191176. doi: 10.1371/journal.pone.0191176 (PMC5774754; doi:10.1371/journal.pone.0191176)

**Supporting Information File 1**

Figure A: Classification tree trained on 2013 and 2014 data and tested on 2012 data


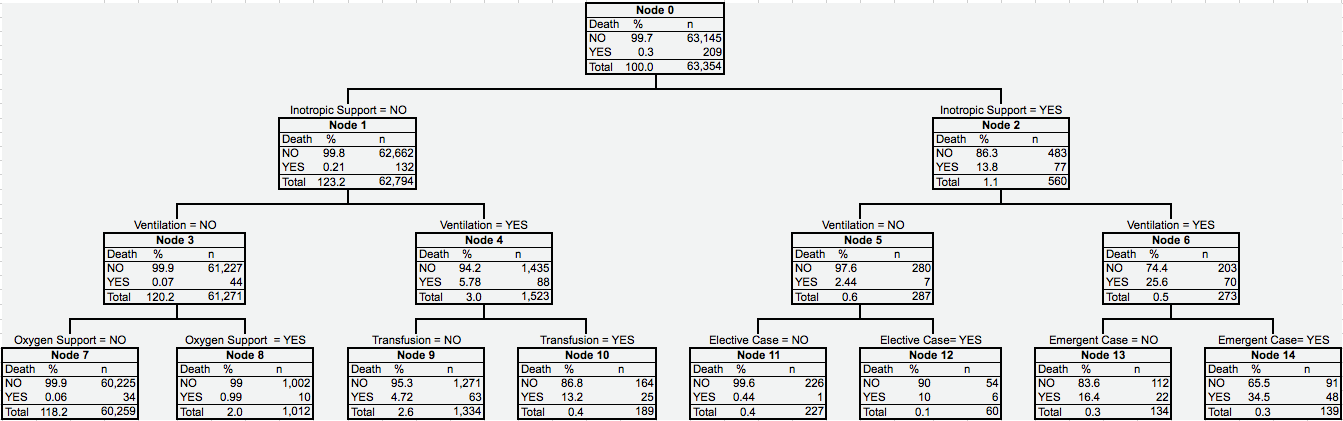


Figure B: Classification tree trained on 2012 and 2014 data and tested on 2013 data


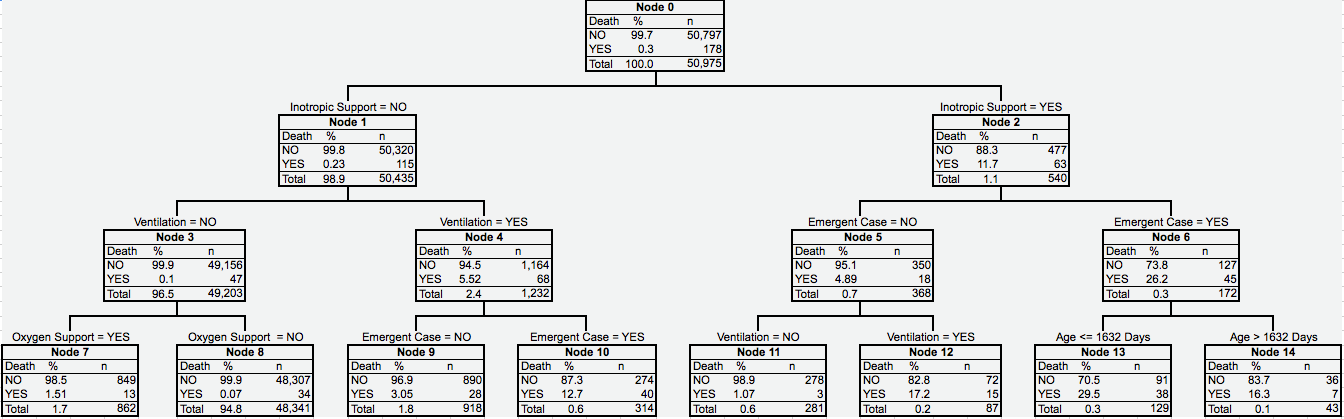


Figure C: Classification tree trained on 2012 and 2013 data and tested on 2014 data
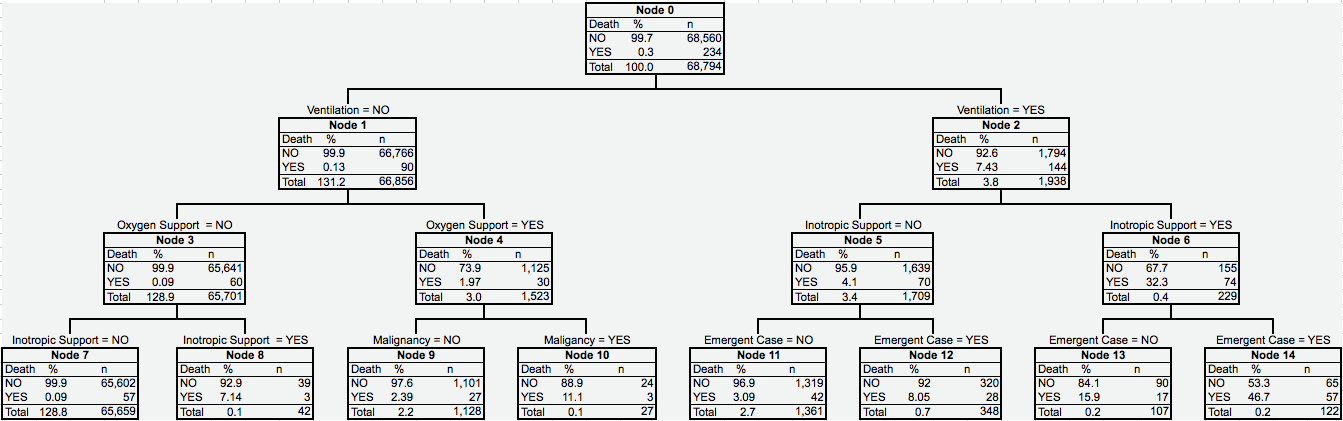

Supplement: S1 File — Figure A: Classification tree trained on 2013 and 2014 data and tested on 2012 data. Figure B: Classification tree trained on 2012 and 2014 data and tested on 2013 data. Figure C: Classification tree trained on 2012 and 2013 data and tested on 2014 data. (DOCX) [file pone.0191176.s003.docx]
