# Supplementary material for: A novel risk classification system for 30-day mortality in children undergoing surgery
Source: PLoS One. 2018 Jan 19;13(1):e0191176. doi: 10.1371/journal.pone.0191176 (PMC5774754; doi:10.1371/journal.pone.0191176)

**Supporting Information File 3**

S1 Figure: Final classification tree built using data from 2012-2014 using optimal stopping rules


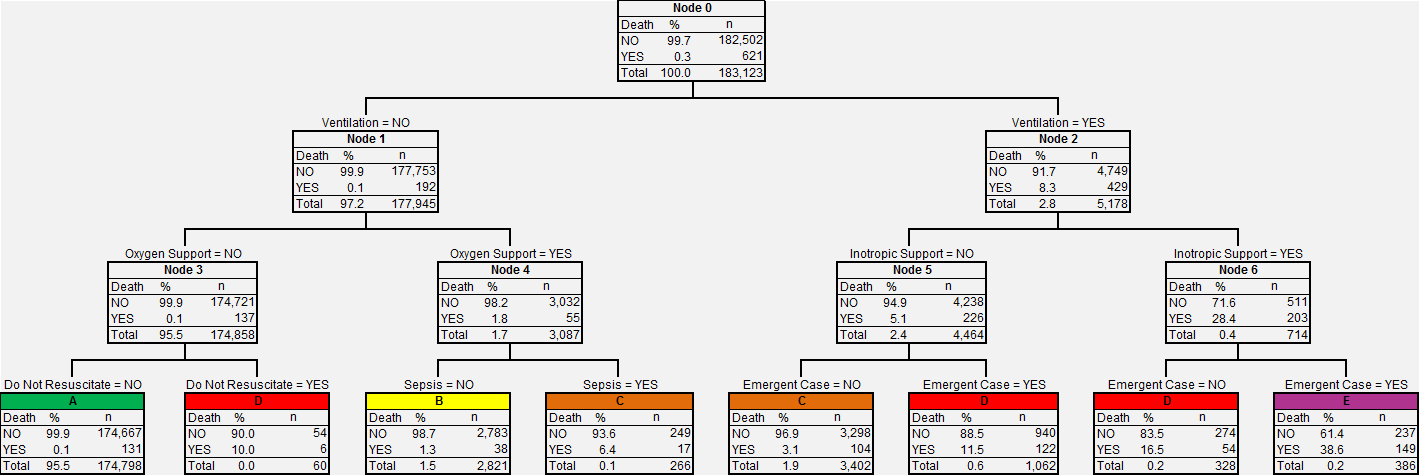

Supplement: S1 Fig — (DOCX) [file pone.0191176.s004.docx]
